# Assessing public knowledge, attitudes and determinants of third COVID-19 vaccine booster dose acceptance: current scenario and future perspectives

**DOI:** 10.1186/s40545-022-00422-2

**Published:** 2022-03-28

**Authors:** Ammar Abdulrahman Jairoun, Sabaa Saleh Al-Hemyari, Faris El-Dahiyat, Maimona Jairoun, Moyad Shahwan, Mena Al Ani, Mustafa Habeb, Zaheer-Ud-Din Babar

**Affiliations:** 1 Health and Safety Department, Dubai Municipality, Dubai, UAE; 2 School of Pharmaceutical Sciences, Universiti Sains Malaysia, 11800 Gelugor, Pulau Pinang Malaysia; 3 Pharmacy Department, Emirates Health Services, Dubai, UAE; 4 College of Pharmacy, Al Ain University, Al Ain, UAE; 5 AAU Health and Biomedical Research Center, Al Ain University, Abu Dhabi, UAE; 6 College of Pharmacy and Health Sciences, Ajman University, Ajman, UAE; 7 Center of Medical and Bio-Allied Health Sciences Research, Ajman University Ajman, Ajman, 346 UAE; 8 College of Medicine, University of Sharjah, Sharjah, UAE; 9 Department of Family and Community Medicine and Behavioural Science University of Sharjah, Sharjah, UAE; 10 Edgware Community Hospital Barnet, Enfield and Haringey Mental Health, NHS Trust, Edgware, UK; 11 Department of Pharmacy, University of Huddersfield, Huddersfield, HD1 3DH UK

**Keywords:** Covid-19, Vaccination, Knowledge, Attitude, Vaccine hesitancy, Acceptance

## Abstract

**Background:**

People with weakened immune systems may not develop adequate protection after taking two doses of the mRNA-combined COVID-19 vaccine. The additional dose may improve the level of protection against Covid-19.

**Objectives:**

Current study aimed to evaluate the knowledge, attitude and determents of third COVID-19 vaccine booster dose acceptance among population in the UAE.

**Methods and materials:**

This is online descriptive cross-sectional community-based study conducted among the students and faculty of Ajman University from 25 August to 20 October 2021. The questionnaire, which was in the English language, encompassed two sections containing 22 items. Section one gathered the demographic details of the respondents, while Section two used 13 questions to evaluate the respondents’ knowledge of and attitude to the third COVID-19 vaccine booster dose.

**Results:**

614 respondents participated in this study. The average knowledge score was 44.6% with a 95% confidence interval (CI) of [41%, 49%]. Better knowledge scores were observed in postgraduates (OR 4.29; 95% CI 2.28–8.11), employees in the healthcare sector (OR 1.62; 95% CI 1.05–2.51), participants who had relatives infected with the Covid-19 (OR 1.46; 95% CI 1.05–2.02), participants who had infected with Covid-19 (OR 2.21; 95% CI 1.43–3.43) and participants who had received first two doses of the COVID-19 vaccine (OR 2.08; 95% CI 1.40–3.11). The average attitude score was 70.2% with a 95% confidence interval (CI) of [69.2%, 71.2%].

**Conclusion:**

Necessary steps should be taken by the government and public health authorities, in line with the local culture, to increase vaccination acceptance and foster positive attitudes towards the vaccine. A suitable approach to this would be to develop an educational framework that could demonstrate the risks of vaccine avoidance or delay to the general population. Moreover, health authorities should pay more attention to the false information being disseminated across the internet, especially social media. Also, healthcare workers should be trained in vaccinology and virology to make sure that they are able to understand important developments in these fields and convey the findings to their patients.

## Introduction

Covid-19 infection caused by Severe Acute Respiratory Syndrome Virus 2 (SARS-CoV-2), resulting in a disease called coronavirus disease 2019 (Covid-19) [1, 2]. In March 2020, the World Health Organization (WHO) announced that it had classified COVID-19 as a pandemic [2]. The virus that causes Covid-19 appears to spread easily between people and scientists will continue to discover more about how it spreads over time, the available data showed that it spreads through close personal contact (within 6 feet, or 2 m) [3]. Additionally, the virus spreads through released respiratory droplets when an infected person coughs, sneezes, breathes, talks, this spray can be inhaled or enter into the mouth, nose, or eyes of a nearby person [4]. However, Covid-19 can sometimes be spread by exposure to small droplets or mists that remain in the air for several minutes or hours. This is called airborne transmission [5]. Signs and symptoms of Covid-19 may appear two to 14 days after exposure [6]. The period after exposure to the virus and before symptoms appear is called the incubation period [7]. Common signs and symptoms may include: fever, cough, tiredness and loss of taste or smell. Other symptoms of the disease include: shortness of breath or difficulty breathing, muscle pain, sore throat, runny nose, headache, chest pain, redness eye (conjunctivitis), nausea, vomiting and diarrhea [6]. Symptoms of Covid-19 can range from mild to severe, as some people may have only a few symptoms, while others have no symptoms at all [8]. Some people may feel worse about a week after they start, such as worsening shortness of breath and pneumonia [9]. The risk of developing severe symptoms of Covid-19 infection increases with age [10]. Also, certain conditions may increase your risk of developing severe symptoms from Covid-19, including: serious heart disease, cancer, chronic obstructive pulmonary disease, having type 1 or type 2 diabetes, obesity, hypertension, smoking, chronic kidney disease, sickle cell disease or thalassemia, pregnancy and asthma [11–13]. The U.S. Food and Drug Administration (FDA) has granted authorization for some COVID-19 vaccines in the United States. FDA has approved many vaccines, to prevent Covid-19 in 16 years and older people [14]. The vaccine can prevent you from infection with COVID-19 or from developing severe illness if you become infected with the virus that causes it. In addition, the COVID-19 vaccine may provide better protection against infection with COVID-19. A recent study showed that unvaccinated people who had previously had Covid-19 were twice as likely to have a recurrence as those who did get vaccinated [15]. The Centers for Disease Control and Prevention (CDC) recommend a booster dose for people age 65 or older, some people who have been fully vaccinated and whose immune response has weakened over time, such as people who had an organ transplant [16, 17]. People with weakened immune systems may not develop adequate protection after taking two doses of the mRNA-combined COVID-19 vaccine. The additional dose may improve the level of protection against Covid-19. The third dose should be given at least 28 days after the second dose of the mRNA vaccine [18].

Due to the rise of vaccine hesitancy, developing safe vaccines and demonstrating their effectiveness is no longer enough to fight a disease. Referring to people’s general uncertainty towards vaccines and their unwillingness to take them, vaccine hesitancy has been listed among the most pressing issues facing public health [19], not least in the context of the COVID-19 pandemic.

In a survey of the general population in Saudi Arabia, 64.7% expressed a willingness to take the COVID-19 vaccine [20]. Another recent study systemically reviewing the extant literature on vaccine acceptance in 33 countries revealed a variation in line with income level and geographical region. For example, Jordan (28.4%) and Kuwait (23.6%), among others, had low acceptance, while Poland (56.3%), Italy (53.7%), and Russia (54.9%) showed moderate acceptance. High acceptance was found to be particularly the case in in eastern Asia, including Malaysia (94.3%), Indonesia (93.3%), and China (91.3%). The conclusion may be drawn that there is a need to enhance the willingness to take the COVID-19 in a number of countries, with the aim of achieving global herd immunity and ending the pandemic [21]. Several positive reasons for vaccine willingness have been noted, such as helping one’s society and improving the country’s health situation, which would strengthen its economy [22, 23]. On the other hand, vaccine hesitancy occurs for reasons such as mistrust or a lack of confidence—either in the government or in the vaccine itself—in addition to absent information, contradicting official recommendations, and vaccine safety and effectiveness issues [24–27]. Fortunately, it seems that vaccine hesitancy is declining as more information on the vaccine’s safety and effectiveness becomes available [24]. However, there are still certain factors affecting individuals’ decision to take the vaccine that need to be addressed, including sociodemographic factors, such as education level, as well as attitudes, political standpoints, and perceptions of COVID-19 [28–32]. Healthcare workers have been found to high vaccine hesitancy, despite their crucial role in fighting the pandemic, whether through modeling good health behavior or actually giving the vaccine. Ref. [33], reviewing 35 studies on healthcare workers and vaccine hesitancy, found a wide range of vaccination hesitancy levels globally, ranging between 4.3% and 72%. Healthcare workers’ perceptions and attitudes towards COVID-19 and its vaccines have been found to be particularly salient to their vaccine hesitancy. Meanwhile, healthcare workers’ role, which puts them first in line to receive the vaccine, as well as their task of administering vaccines to the rest of the population, means that the influential factors shaping their attitudes towards the vaccine, e.g., knowledge, urgently need to be investigated [34]. According to a study in the UAE, people with Arabic nationalities were less likely to take a COVID-19 vaccine compared to the rest of the population, while health science students were more likely to get the vaccine than non-health science students. While a vaccine acceptance rate of 56.3% was found among the study participants, they still reported concerns about unforeseen problems (65.5%) and unforeseen side effects (35.1%) as well as general mistrust (47.3%). The study also found that the participants’ knowledge of COVID-19 varied significantly [35]. Despite the plentiful vaccination opportunities in the UAE, the novelty of the vaccines themselves is highlighting the issues of vaccine acceptance in the country. Furthermore, there is currently a gap regarding knowledge, perceptions and attitudes towards the COVID-19 vaccine among the general population. To address this gap, this novel work examines the knowledge, perceptions and attitudes towards the COVID-19 vaccine in the UAE, specifically the willingness to receive the third COVID-19 booster dose.

## Materials and methods

### Study design and setting

This descriptive, cross-sectional analytical study was performed on the community of the faculty and students at Ajman University (AU). The aim was to evaluate their knowledge of and attitude towards receiving a third COVID-19 vaccine dose (hereafter, COVID-19 vaccine booster). Potential respondents were sent an online link via email, and the data collection period was from 25 August to 20 October 2021.

### Study participants (inclusion and exclusion criteria)

The target population comprised the students and faculty of AU, including UAE nationals as well as resident non-nationals. The following were used as inclusion criteria: (1) aged 18 years and above and (2) willing to participate in the study.

### Questionnaire design

We performed a pilot study in AU towards the end of August 2021 as no work has thus far examined this topic in the context of the UAE. Previous studies exploring knowledge of and attitude towards the COVID-19 vaccine [36–38] were drawn upon to develop the pilot questionnaire. Subsequently, we constructed a self-administered questionnaire based on pre-existing surveys; hereby, all key points of the subject under study were incorporated and the questionnaire was tailored to fit the UAE context. Experts in the subject were asked to review and evaluate the design, content, and relevance of the questionnaire as well as assess its comprehensibility and readability. Three lecturers in pharmacy at AU were then asked to validate the questionnaire, which was consequently modified slightly based on their feedback. The pilot study was then performed on 55 participants using the designed survey before it was fully implemented on the study population.

The questionnaire’s quantitative content validity was estimated by calculating Lawshe’s content validity ratio (CVR) [39] for each item. All items that scored at least 0.78 were considered acceptable; those not achieving the threshold value of 0.78 were removed from the final questionnaire [39]. Next, the mean CVR value of the retained items was calculated to obtain the content-validity index (CVI). The CVI of 0.85 indicated that the questionnaire’s final validity is acceptable [40]. The reliability of the survey was assured through the modifications based on the results from the abovementioned pilot study. Furthermore, as the pilot study raised no issues with the survey instrument, it was used in the principal study, with the abovementioned minor modifications. The pilot study participants were not included in the final analysis. The reliability of the questionnaire was further ensured by calculating Cronbach’s α; the α-value of 0.75 demonstrated that the internal consistency is acceptable.

The questionnaire was in English and incorporated 22 items in two sections. The items in section one collected the demographic details of the respondents, such as age, sex, employment status and level of education. The 13 items in section two aimed to capture the respondents’ knowledge of and attitude towards the COVID-19 vaccine booster.

### Questionnaire scoring

The respondents’ knowledge of the COVID-19 vaccine booster was measured using one item, namely the question “Do you know about the third COVID-19 vaccine booster dose?” Possible answers for this item were “yes” (scoring “1”) and “no” or “don’t know” (both scoring “0”). The remaining 12 items assessed the respondents’ attitudes towards the COVID-19 vaccine booster using a 5-point Likert scale (0 = “strongly disagree”, 1 = “disagree”, 2 = “neutral”, 3 = “agree” and 4 = “strongly agree”). The grading of the 12 items was achieved by summing the raw Likert-scale scores for each respondent. Based on this, a percentage between 0 and 100% was calculated for each respondent, reflecting their general attitude towards the COVID-19 vaccine booster.

### Sample size and sampling technique

As highlighted above, a pilot study was used to estimate a suitable sample size for the final survey. Hereby, the pilot questionnaire was sent via email to the target population, i.e., the students and faculty at AU (*n* = 100); 55 responses were received, giving a response rate of 55%. The calculation of the sample size for the final survey was based on the pilot respondents’ answers to the question “Do you know about the third COVID-19 vaccine booster dose?”, to which around 50% responded “yes”. The selected alpha level of 5% meant a confidence interval (CI) of 95%. The precision (*D*) of the 95% CI was set to 5%, giving a maximum width of 10% for the 95% CI. Thus, assuming a non-response rate of 40%, a sample size of 640 participants was considered to be suitable. Potential respondents were contacted with the assistance of the Admission and Registration Department at AU, which provided an Excel spreadsheet listing the names, colleges, study years, and email addresses of the students and faculty at AU. Simple random sampling based on the identification (ID) number was used to select potential respondents. Thereafter, the pre-selected respondents were stratified according to their department and college.

### Questionnaire administration

The self-administered questionnaire was sent to the randomly selected potential respondents from AU via an online link sent to their emails. On the questionnaire itself, the first page clarified the study’s nature and purpose. Respondents were considered to have consented to participate if they proceeded to the next page. Non-respondents were sent reminder emails every month, and all respondents who completed the study received a thank-you message in an email. No incentives of any kind were offered to the respondents in return for their completing the survey.

### Ethical considerations

The Institutional Ethical Review Committee of AU approved this study. All individuals who participated in the survey did so voluntarily. The study purpose was outlined on the cover page of the questionnaire, and respondents’ continuing to the subsequent page were assumed to have given their consent. Respondents’ identities were not recorded in any way, and they were assured of their confidentiality.

### Statistical analysis

The statistical analysis was performed via the Statistical Package for the Social Sciences software version 24 (SPSS; IBM Corp., Armonk, NY, USA). Percentage and frequency were used to present the qualitative variables, while the quantitative variables were presented as mean ± standard deviation (SD). The correlations between knowledge of the COVID-19 vaccine booster and the demographic factors were assessed using a Chi-square test. Differences across groups in the quantitative variables were evaluated using unpaired Student’s t-tests, non-parametric versions, and one-way ANOVA. To investigate the factors affecting respondents' attitude and willing to get the third COVID-19 booster dose, the median score was calculated by categorize the attitude score into dichotomous/binary outcome. In doing so, 34 was calculated as the median score. This dichotomous outcome variable mentioned above was then used in logistic regression models. Variable selection and model building were achieved using the stepwise method. Statistical significance was assumed for *p*-values below 0.05.

## Results

### Demographic and baseline characteristics

The demographic factors of the participants are presented in Table 1. A total of 614 respondents participated in this study. Among the total, 69.1% (*n* = 424) were female and 30.9% (*n* = 190) male. The age of the participant was detailed as follows: 191 (31.1%) aged 18–22, 236 (38.4%) aged 23–26, 131 (21.3%) aged 27–30, 30 (4.9%) aged 31–36 and 26 (4.2%) aged ≥ 37. Of the total, 22.1% were Emirati, 22.1% were African, 19.7% were Western, 28% were Asian and 8% were Arabic. The educational levels reported were 118 (19.2%) had primary school/elementary, 195 (31.8%) had secondary education, 162 (26.4%) had diploma, 66 (10.7%) were university degree holders and 73 (11.9%) were postgraduates. Among the total subjects, 24.3% (*n* = 149) were students, 19.5% (*n* = 120) were unemployed, 26.5% (*n* = 163) were employee in health sector and 29.6% (*n* = 182) were employee in non-health sector. Of the total participants, 11.7% have chronic diseases, 37.5% have relatives who infected with the Covid-19, 16.3% have infected with Covid-19, 77% have received first two doses of the COVID-19 vaccine and 10.3% have admitted to hospital due to Covid-19 symptoms.

**Table 1:** Demographic characteristics of study participants (*n* = 614)

Demographic	Groups	Frequency	Percentage (%)
Gender	Male	190	30.9
	Female	424	69.1
Age	18–22	191	31.1
	23–26	236	38.4
	27–30	131	21.3
	31–36	30	4.9
	≥ 37	26	4.2
Nationality	Emirati	136	22.1
	African	136	22.1
	Western	121	19.7
	Asian	172	28
	Arabic	49	8
Education	Primary school/elementary	118	19.2
	Secondary education	195	31.8
	Diploma	162	26.4
	University degree	66	10.7
	Post-graduate degree	73	11.9
Employment	Student	149	24.3
	Unemployed	120	19.5
	Employee in health sector	163	26.5
	Employee in non-health sector	182	29.6
Do you have any chronic diseases	Yes	72	11.7
	No	542	88.3
Do you have any of your relatives who infected with the Covid-19	Yes	230	37.5
	No	384	62.5
Have you ever infected with Covid-19	Yes	100	16.3
	No	514	83.7
Have you received first two doses of the COVID-19 vaccine	Yes	473	77
	No	141	23
Have you admitted to hospital due to COVID-19 symptoms	Yes	63	10.3
	No	551	89.7

### Participant’s knowledge about third COVID-19 vaccine booster dose

The average knowledge score was 44.6% with a 95% confidence interval (CI) of [41%, 49%]. The knowledge about third COVID-19 vaccine booster dose was evaluated by asking the participants “Do you know about third COVID-19 vaccine booster dose.”

Table 2 displays the results of logistic regression analysis for the factors influence the knowledge about third COVID-19 vaccine booster dose. The results of this procedure showed that better knowledge scores were observed in postgraduates (OR 4.29; 95% CI 2.28–8.11), employees in the healthcare sector (OR 1.62; 95% CI 1.05–2.51), participants who had relatives infected with the Covid-19 (OR 1.46; 95% CI 1.05–2.02), participants who had infected with Covid-19 (OR 2.21; 95% CI 1.43–3.43) and participants who had received first two doses of the COVID-19 vaccine (OR 2.08; 95% CI 1.40–3.11).

**Table 2:** Participants knowledge according to demographic variables

Demographic	Groups	Estimate (%)	Participants knowledge			
			OR	95% CI		*P*-value
				Lower	Upper	
Gender	Male	88 (46.3)	Ref.			
	Female	186 (43.9)	0.91	0.64	1.28	0.57
Age	18–22	78 (40.8)	Ref.			
	23–26	110 (46.6)	1.27	0.86	1.86	0.23
	27–30	60 (45.8)	1.22	0.78	1.92	0.38
	31–36	15 (50)	1.45	0.67	3.13	0.35
	≥ 37	11 (42.3)	1.06	0.46	2.44	0.89
Nationality	Emirati	58 (42.6)	Ref.			
	African	61 (44.9)	1.09	0.68	1.77	0.71
	Western	44 (36.4)	0.77	0.46	1.27	0.30
	Asian	82 (47.7)	1.23	0.78	1.93	0.38
	Arabic	29 (59.2)	1.95	1.01	3.78	0.05
Education	Primary school/elementary	45 (38.1)	Ref.			
	Secondary education	84 (43.1)	1.23	0.77	1.96	0.39
	Diploma	65 (40.1)	1.09	0.67	1.77	0.74
	University degree	27 (40.9)	1.12	0.61	2.08	0.71
	Post-graduate degree	53 (72.6)	4.29	2.28	8.11	< 0.001*
Employment	Student	64 (43.0)	Ref.			
	Unemployed	48 (40.0)	0.88	0.54	1.44	0.63
	Employee in non-health sector	62 (38.0)	0.82	0.512	1.28	0.38
	Employee in health sector	100 (54.9)	1.62	1.05	2.51	0.03*
Do you have any chronic diseases	Yes	33 (45.8)	1.06	0.65	1.73	0.83
	No	241(44.5)	Ref.			
Do you have any of your relatives who infected with the Covid-19	Yes	116 (50.4)	1.46	1.05	2.02	0.025*
	No	158 (41.1)	Ref.			
Have you ever infected with Covid-19	Yes	61 (61)	2.21	1.43	3.43	< 0.001*
	No	213 (41.4)	Ref.			
Have you received first two doses of the COVID-19 vaccine	Yes	230 (48.6)	2.08	1.40	3.11	< 0.001*
	No	44 (31.2)	Ref.			
Have you admitted to hospital due to COVID-19 symptoms	Yes	43 (68.3)	2.98	1.71	5.19	< 0.001*
	No	231 (41.9)	Ref.			

*P*-values less than 0.05 were considered statistically significant, OR, odds ratio; CI, confidence interval

### Participant’s attitude about the third COVID-19 vaccine booster dose

The average attitude score was 70.2% with a 95% confidence interval (CI) of [69.2%, 71.2%]. The attitude towards third COVID-19 vaccine booster dose was evaluated by asking the participants 12 questions.

Table 3 shows the attitude about third COVID-19 vaccine booster dose according to demographics. Among the studied variables, the univariate analysis revealed that gender (*P* = 0.002), nationality (*P* = 0.01), education (*P* = 0.001) Employment status (*P* = 0.001), having chronic diseases (*P* = 0.001), having relatives infected with the Covid-19 (*P* < 0.001), being infected with Covid-19 (*P* < 0.001), received first two doses of the COVID-19 vaccine (*P* < 0.001) and being admitted to hospital due to COVID-19 symptoms (*P* < 0.001) were associated with the attitude towards third COVID-19 vaccine booster dose.

**Table 3:** Participants attitude about the third COVID-19 vaccine booster dose according to demographic variables

Demographic	Groups	Participants attitude			
		Mean ± SD		Median	*P*-value
Gender	Male	69.243	0.895	70.86	0.207
	Female	70.602	0.599	70.81	
Age	18–22	70.910	0.891	70.44	0.138
	23–26	69.677	0.801	70.34	
	27–30	68.655	1.075	70.81	
	31–36	73.819	2.247	79.2	
	≥ 37	72.917	2.414	70.88	
Nationality	Emirati	71.829	1.043	70.80	0.01*
	African	67.907	1.043	68.7	
	Western	67.269	1.106	67.5	
	Asian	71.669	0.928	71.8	
	Arabic	73.895	1.738	75	
Education	Primary school/elementary	72.087	1.024	71.8	0.001*
	Secondary education	69.701	0.797	70.87	
	Diploma	65.985	0.874	68.8	
	University degree	64.173	1.369	70.8	
	Post-graduate degree	83.134	1.302	85.4	
Employment	Student	53.929	0.458	56.2	0.001*
	Unemployed	66.719	0.511	66.6	
	Employee in non-health sector	72.699	0.438	72.9	
	Employee in health sector	83.516	0.415	81.3	
Do you have any chronic diseases	Yes	60.590	1.395	60.4	0.001*
	No	71.456	0.509	70.8	
Do you have any of your relatives who infected with the Covid-19	Yes	81.739	0.560	79.2	< 0.001*
	No	63.260	0.434	66.7	
Have you ever infected with Covid-19	Yes	88.000	0.951	85.4	< 0.001*
	No	66.715	0.420	68.7	
Have you received first two doses of the COVID-19 vaccine	Yes	71.921	0.548	70.83	< 0.001*
	No	64.347	1.004	66.6	
Have you admitted to hospital due to COVID-19 symptoms	Yes	91.534	1.261	89.6	< 0.001*
	No	67.740	0.426	70.8	

^*^P-values less than 0.05 were considered statistically significant, P-values obtained from the Kruskal–Wallis and Mann–Whitney U tests

Table 4 shows the results of each questions related to the attitude towards third COVID-19 vaccine booster dose,

**Table 4:** Participants’ attitude about the third COVID-19 vaccine booster dose

Attitude items	Strongly disagree		disagree		Neutral		agree		Strongly agree	
	*F*	%	*F*	%	*F*	%	*F*	%	*F*	%
Third vaccine dose (COVID-19 vaccine booster) help increase your immunity against the virus	2	0.3	19	3.1	75	12.2	165	26.9	353	57.5
Is it very dangerous for health using third dose vaccine (COVID-19 vaccine booster)? (reverse scored)	181	29.5	204	33.2	124	20.2	60	9.8	45	7.3
Third vaccine dose (COVID-19 vaccine booster) increase allergic reactions. *(reverse scored)*	118	19.2	195	31.8	162	26.4	66	10.7	73	11.9
Third vaccine dose (COVID-19 vaccine booster) increase autoimmune diseases. (reverse scored)	65	10.6	162	26.4	161	26.2	138	22.5	88	14.3
The newly third vaccine dose (COVID-19 vaccine booster) is safe and effective	2	0.3	17	2.8	77	12.5	198	32.2	320	52.1
The third vaccine dose (COVID-19 vaccine booster) is essential for UAE	9	1.5	30	4.9	111	18.1	189	30.8	275	44.8
I will encourage my family, friends and relatives to get the third vaccine dose (COVID-19 vaccine booster)	9	1.5	23	3.7	71	11.6	181	29.5	330	53.7
I will take third vaccine dose (COVID-19 vaccine booster) without any hesitation, if it is available in my country	6	1.0	34	5.5	52	8.5	111	18.1	411	66.9
It is not possible to reduce the prevalence of COVID-19 without a third vaccine dose (COVID-19 vaccine booster)	8	1.3	31	5.0	124	20.2	168	27.4	283	46.1
I worry about serious unknown long-term effects of the third vaccine dose (COVID-19 vaccine booster) in the future	31	5.0	103	16.8	198	32.2	152	24.8	130	21.2
I believe that third vaccine dose (COVID-19 vaccine booster) make a lot of money for pharmaceutical companies *(reverse scored)*	11	1.8	47	7.7	149	24.3	233	37.9	174	28.3
I believe that first two doses of the COVID-19 vaccine gives the safest protection *(reverse scored)*	29	4.7	97	15.8	172	28.0	199	32.4	117	19.1

*F*, frequency; %, percentage

### Factors influencing the attitude towards third COVID-19 vaccine booster dose

Table 5 displays the of multivariate regression model applied to demographic factors. To select the set of the factors that jointly influence the attitude towards third COVID-19 vaccine booster dose. The results of this procedure showed that better attitude towards third COVID-19 vaccine booster dose were observed in unemployed participants (OR 2.47; 95% CI 2.14–2.86), employee in non-health sector (OR 2.052; 95% CI 1.89–2.23), employee in health sector (OR 1.611; 95% CI 1.49–1.74), participants who had relatives infected with the Covid-19 (OR 1.183; 95% CI 1.057–1.323), those had infected with Covid-19 (OR 1.25; 95% CI 1.08–1.44), those who received first two doses of the COVID-19 vaccine (OR 1.08; 95% CI 1.02–1.15) and those who had admitted to hospital due to COVID-19 symptoms (OR 2.25; 95% CI 1.84– 2.74).

**Table 5:** Multivariate analysis of factors associated with the attitude about third COVID-19 vaccine booster dose

Demographic	Groups	Attitude score ≥ 34			
		OR	95% CI		*P*-value
			Lower	Upper	
Gender	Male	I			
	Female	0.990	0.934	1.050	0.743
Age	18–22	1.024	0.881	1.190	0.760
	23–26	1.028	0.884	1.194	0.723
	27–30	0.998	0.856	1.162	0.975
	31–36	0.926	0.764	1.121	0.431
	≥ 37	I			
Nationality	Emirati	1.032	0.905	1.178	0.636
	African	1.020	0.901	1.155	0.752
	Western	0.929	0.823	1.050	0.239
	Asian	0.999	0.894	1.118	0.991
	Arabic	I			
Education	Primary school/elementary	0.933	0.820	1.062	0.295
	Secondary education	0.905	0.798	1.026	0.120
	Diploma	0.892	0.787	1.011	0.074
	University degree	0.888	0.775	1.019	0.091
	Post-graduate degree	I			
Employment	Student	I			
	Unemployed	2.473	2.138	2.860	< 0.001*
	Employee in non-health sector	2.052	1.891	2.227	< 0.001*
	Employee in health sector	1.611	1.489	1.743	< 0.001*
Have any chronic diseases		0.876	0.801	0.959	0.004*
Have relatives who infected with the Covid-19		1.183	1.057	1.323	0.003*
Have infected with Covid-19		1.246	1.079	1.440	0.002*
Received first two doses of the COVID-19 vaccine		1.080	1.015	1.148	0.015*
Admitted to hospital due to COVID-19 symptoms		2.249	1.843	2.744	< 0.001*

^*^P-values less than 0.05 were considered statistically significant, OR, odds ratio; CI, confidence interval

## Discussion

The COVID-19 vaccines have been proven to be safe and effective against the disease, and governments across the world have been putting in efforts to relay this information to their populations. Nonetheless, there remain barriers to the public’s willingness to receive the vaccine. To effectively fight against the pandemic, it is necessary to stop the transmission of the virus through herd immunity, which requires a vaccination rate of at least 82% and, hence, strong willingness to vaccinate and low vaccine hesitancy [41]. This shows the importance of determining which factors influence individuals’ vaccine hesitancy and acceptance, which will allow important policy changes to be made and enable public health authorities to develop conceptual frameworks and campaigns that can educate the general population, raising their awareness of the importance of taking the vaccine [42]. As yet, there has been an absence of published evidence on the UAE population’s knowledge, perceptions and attitudes towards the booster dose.

We found that only 44.6% of the participants knew about the booster dose, meaning that more than half of the participants had a low knowledge level on this issue. A substantial body of prior research has established a similar lack of knowledge about COVID-19 vaccines [35, 43–45]. In the present study, this lack of knowledge may be linked to the lack of information dissemination by the government among the public on the importance of taking the vaccine, particularly the booster dose. Meanwhile, the severity of COVID-19, including its mortality rates, may have been underreported or incorrectly transmitted, thereby mitigating the urgency to get the vaccine and highlighting the questions of its safety [46], while the UAE population may be more hesitant to inform themselves about the disease or its vaccines. This underlines the need to give the members of the community access to information on the vaccines that is trusted and grounded in solid evidence. When designing vaccination education campaigns, policymakers should seek to enhance the public’s trust in the vaccines and eliminate the social and financial barriers to vaccination, while aiming to address the public health issues underlying vaccine hesitancy [43–45].

This study found that participants with a higher education level, with a prior history of COVID-19 infection, and a previous experience of having taken the vaccine all demonstrated more knowledge of the booster dose. This is in line with the findings of prior research [47–50]. Similarly, research in China [51] and Hong Kong [48] has revealed that individuals who had already taken the influenza vaccine showed a higher willingness to take the COVID-19 vaccine. This is likely to be due to having had a prior positive experiences of receiving a vaccine.

Overall, 70.2% of the study’s participants expressed that they would receive the booster dose. In comparison, the literature has found that vaccine hesitancy rates range widely across populations and countries. For example, Lazarus et al., as part of a global survey, identified vaccine acceptance rates between below 55% (Russia) and 90% (China) [52]. Similarly, Sallam et al. [53], in a literature review, found substantial variation in vaccine acceptance rates among adults, reaching a high of 97% in Ecuador and lows of 23.6% in Kuwait and 28.4% [23]. Research in the context of sub-Saharan Africa indicated 51% vaccine acceptance, while 67% was found for Saudi Arabia [39]. Finally, Sallam, performing a systemic review, further found vaccine acceptance rates ranging from 27.7% (Congo) to 78.1% (Israel) [54, 55]. The findings for vaccine acceptance among healthcare workers are similar, with substantial variation seen among health science students globally and increased vaccine hesitancy levels in middle- and low-income nations [56, 57].

Recent research in the UAE found that the vaccine acceptance rate was influenced by concerns about unforeseen problems (65.5%), unforeseen impacts (35.1%) and general mistrust (47.3%) [35]. These concerns tended be linked to the potential side effects of the vaccine as well as the limited availability of trial data assessing its benefits. This finding highlights the need to implement stronger and more effective health education efforts that prioritize a wide range of sociodemographic categories, with the ultimate aim of enhancing the uptake of COVID-19 vaccination, both in the UAE and globally. More information on the vaccines’ side effects and effectiveness would be of great benefit as the research has demonstrated that increased knowledge leads to reduced vaccine hesitancy rates in the long term [32, 58, 59]. A suitable way to achieve this is by strengthening public health education and employing vaccine promoters, such as healthcare workers, to increase the public’s willingness to take the vaccine [60–62].

The findings of this study reveal that individuals’ attitudes towards and willingness to take the booster dose are influenced by a significant number of sociodemographic factors. As a result, we expect that our results will be able to inform future campaigns aiming to foster awareness of health in general and of COVID-19 vaccination in particular.

We found that participants who had a higher education and who had already received both COVID-19 vaccine doses early on were more positively inclined towards COVID-19 vaccination in general and were more willing to take the booster dose. This is consistent with the previous research [31, 38, 48, 49, 51, 63]. For example, a study in Saudi Arabia found that individuals working in the public sector and who had at least a postgraduate degree were more willing to take the COVID-19 vaccine [20].

Furthermore, those participants in our study who had chronic health issues were significantly more likely to be amenable to the booster dose. This contradicts a previous finding that revealed that participants who have chronic diseases are significantly less likely to accept the booster dose than healthy individuals [45]. COVID-19 patients with underlying conditions, such as hypertension, cancer, cardiovascular disease, congestive heart failure, diabetes, and chronic kidney disease, have a significantly higher risk of mortality upon contracting the disease [64], making it crucial for this population to receive the vaccine as soon as possible. As a result, they should be prioritized, and more efforts must be made to raise their awareness of the severity and implications of COVID-19 and how the vaccine can mitigate these.

Among the healthcare workers surveyed in this study, 83.5% expressed that they intended to be vaccinated and 16.5% explicitly stated they would not take the vaccine. A previous study in South and Southeast Asia found high vaccine acceptance rates (at least 95%) in that region, with healthcare workers showing a high willingness to receive the vaccine [65]. This was attributed to their perception of the severity of COVID-19, the safety of the vaccines, the lack of financial constraints, low vaccination stigmatization, and a high level of trust in the public health authorities [65]. Meanwhile, vaccine acceptance was previously found to be in 76.63% in China [66] and 76.10% in Vietnam [67], which is somewhat below the rate found in this study. The conclusion could be drawn that these high rates were due to the participants’ good knowledge of the disease’s severity, trust in the effectiveness and safety of the vaccines, and the fact that the study took place earlier on in the pandemic than our study. A study in Iraq found a lower rate of vaccine acceptance (61.7%) [68], although this was a higher rate than previous work has found for the US; here, above half the surveyed healthcare workers expressed that they had not yet made a decision on the matter [69, 70]. There have been similar findings of low acceptance rates across the world, including in Ghana (39.3%) [71]), Nepal (38.3%) [72], the Democratic Republic of Congo (27.7%) [73], and Egypt (21%) [74], while the research has found rates of 50.2% in Nigeria [75]and 50.52% in Saudi Arabia [76].

A low willingness to take the vaccine could be linked to healthcare workers’ concerns about the vaccines’ efficacy, safety, and side effects, in addition to low vaccine confidence, a lack of knowledge, and mistrust in the vaccines’ effectiveness [63, 77, 78]. Meanwhile, higher vaccine acceptance has been found for individuals with higher socioeconomic status, those who are in direct contact with patients as part of their profession, a perception of risk and fear of the disease, and having previously had an influenza vaccination [33]. Hesitancy among healthcare workers regarding the COVID-19 vaccine could have a two-sided effect: Not only can unvaccinated healthcare workers transmit the virus to their patients, but they are also not be able to encourage their vulnerable patients to receive the vaccine [33].

The study is subject to a few limitations. First, we used a cross-sectional survey, which does not allow strong conclusions to be drawn about the possible relationships between the variables; hence, this area would benefit from a longitudinal study. Second, the online self-reporting method used here may have introduced bias in relation to social acceptability and memory; furthermore, older individuals or those with a lower socioeconomic status may have been excluded due to a lack of Internet access. Hence, the results may not be generalizable. Third, some important concerns may have gone unaccounted for because we used a closed-ended questionnaire. Nonetheless, selection, social desirability, and recall biases are sometimes found in observational methodologies, and these may have been reduced in this study due to the simple random sampling technique. In addition, this study is the first to examine community perceptions in the UAE towards the booster dose, meaning that the findings offer important implications for the makers and planners of health policy, with the aim of vaccinating as many people as possible to end the pandemic.

## Conclusions

To the authors’ best knowledge, this study is the first to examine the knowledge, perceptions, and attitudes among the general population in Gulf Cooperation Council countries (GCC) and UAE regarding the COVID-19 vaccine and their willingness to receive the booster dose. We have found that while the participants had generally inadequate knowledge about the booster dose, they did show more positive attitudes towards the vaccination in the context of the UAE. Hence, necessary steps should be taken by the government and public health authorities, in line with the local culture, to increase vaccination acceptance and foster positive attitudes towards the vaccine. A suitable approach to this would be to develop an educational framework that could demonstrate the risks of vaccine avoidance or delay to the general population. Specifically, in order to fight the disease, a transparent educational effort underlining the social benefits of receiving the vaccine would be highly beneficial. Our findings on the public’s knowledge, perceptions and attitudes regarding the booster dose and their refusal to take it can help shape health policies and vaccination campaigns in the UAE. By revealing those factors that are most strongly influencing the willingness to get vaccinated, this study may thus add to the development of a pathway out of the pandemic. Specifically, health authorities should pay more attention to the false information being disseminated across the internet, especially social media. Also, healthcare workers should be trained in vaccinology and virology to make sure that they are able to understand important developments in these fields and convey the findings to their patients.

## Data Availability

The data that support the findings of this study are available from the corresponding author upon reasonable request.
